# Analysis of Th17 and Tc17 Frequencies and Antiviral Defenses in Gut-Associated Lymphoid Tissue of Chronic HIV-1 Positive Patients

**DOI:** 10.1155/2015/395484

**Published:** 2015-06-28

**Authors:** Gabriella d'Ettorre, Giancarlo Ceccarelli, Mauro Andreotti, Carla Selvaggi, Noemi Giustini, Sara Serafino, Ivan Schietroma, Giuseppe Nunnari, Guido Antonelli, Vincenzo Vullo, Carolina Scagnolari

**Affiliations:** ^1^Department of Public Health and Infectious Diseases, University of Rome “Sapienza”, 00185 Rome, Italy; ^2^Pasteur Institute-Cenci Bolognetti Foundation, 00161 Rome, Italy; ^3^Department of Therapeutic Research and Medicines Evaluation, Italian Institute of Health, 00161 Rome, Italy; ^4^Department of Molecular Medicine, Laboratory of Virology, “Sapienza” University of Rome, 00185 Rome, Italy; ^5^Department of Clinical and Molecular Biomedicine, Division of Infectious Diseases, University of Catania, 95122 Catania, Italy

## Abstract

The complex relationship between both the Th1/Th17 and Tc1/Tc17 axis and innate defences in the intestinal mucosa during HIV-1 infection has not been well characterized. This study examined the frequency, phenotype, and functional status of T cell populations in the gut-associated lymphoid tissue and peripheral blood of virologically suppressed HIV-1-infected patients on therapy, focusing on the Th1, Th17, Tc1, and Tc17 cell subsets. We found a persistent immune cell activation (CD38 and HLADR expression) into the GALT despite the higher levels of Th17 and Tc17 in respect to peripheral blood. An upregulation of type I IFN response in GALT compared to the peripheral blood compartment was also recorded. Furthermore, IFN-*α*/*β* levels were negatively related to the frequencies of Th1 naïve cells and Tc1 cell subsets (naïve, central memory, and effector memory) in the GALT. In contrast, no relationships between type I IFN response and Th1 or Tc1 cell subsets in peripheral blood compartment and between IFN-*α*/*β* and Th17/Tc17 in both GALT and peripheral blood district were recorded. These data indicate that prolonged antiretroviral treatment improves GALT immune function despite the persistence of immune activation and type I IFN response in chronic HIV-1 positive patients.

## 1. Introduction

IL-17-producing CD4^+^ T cells (Th17) are known to regulate the permeability of gut mucosa and microbial translocation. T helper type 17 cells mediate a variety of inflammatory reactions through their selective secretion of IL-17A, IL-17F, and IL-22.

Recent studies have demonstrated that Th17 cells are depleted during HIV/SIV infections, suggesting that cell depletion may accelerate the progression to AIDS [1, 2]. The early initiation of antiretroviral therapy (ART) has been reported to preserve the number and function of Th17 with consequent control of HIV-related immune activation [1–3]. Accordingly, HIV-1-infected individuals receiving ART undergo effective CD4^+^ T cell restoration and this is associated with enhanced CD4^+^ Th17 cell accumulation without complete restoration [4]. Furthermore, although IL-17-secreting CD4^+^ and CD8^+^ T cells have been reported, very little is known about the CD8^+^ T subset in HIV-1 infection and its relationship with immune response activation.

A variety of signals can block Th17 commitment including IFN-*γ*, IL-4, and IL-12 [5]. Furthermore, Harrington et al. showed that the CD4^+^ T cell effector cytokine IFN-*γ* potently suppressed the development of the IL-17-producing effector T cells from naïve CD4^+^ precursor cells, providing a mechanism by which the T helper type 1 (Th1) developmental program “antagonizes” the Th17 developmental program and contributes to lineage divergence. Interestingly, IFN-*α*/*β* was also demonstrated to have a negative impact on Th17 development in mice [6], recently extended to human Th17 cells [7]. Therefore, the dysregulated Th17 response during HIV-1 infection could in part be explained by the reported ability of type I IFN to suppress Th17 development [8, 9]. In this regard, the mucosal antiviral responses appeared to be largely type I IFN-driven and included factors that inhibit HIV-1 budding (tetherin/BST2) and infectivity (APOBEC3G) [10]. However, no studies on the relationships between Th17 and type I IFN response have been performed so far. Besides the well-established detrimental effects of type IFN in chronic HIV-1-infected patients, this complex family of cytokines also seems to regulate the effector and memory T cell functions [5, 11, 12].

To our knowledge, this is the first study to evaluate the relationship between type I IFN activation and IFN-*γ* or IL-17A expressing CD3^+^CD4^+^ or CD3^+^CD8^+^ T cell subsets in the gut-associated lymphoid tissue (GALT) of HIV-1-infected subjects. The objectives of the study were to compare the gene expression of IFN-*α*, IFN-*β*, and IFN receptor (R1) between peripheral blood cells and GALT collected from HIV-1-infected patients who achieved a virological suppression in response to ART therapy. In addition, the relationships between activation of type I IFN response and IFN-*γ* or IL-17A-producing CD4^+^ or CD8^+^ (Tc17) T cell subsets (naïve, central memory, and effector memory) were evaluated.

## 2. Methods

### 2.1. Patients

From May to July 2014, ten HIV-1 positive patients successfully treated with ART were recruited at the Division of Infectious Diseases, Department of Public Health and Infectious Diseases, Hospital of “Sapienza” University of Rome (Italy).

The study was approved by the institutional review board (Department of Public Health and Infectious Diseases, “Sapienza” University of Rome and the Ethics Committee of Umberto I General Hospital, Rome). All study participants gave written informed consent.

### 2.2. Laboratory Procedures, Data Collection, and Analysis Sampling

Patients were sampled for peripheral blood (20 mL) and underwent endoscopic procedures. Colonic washing was carried out by PEG administration 24 hours before the examination. The endoscopic procedure was performed with conscious sedation (midazolam 5 mg/iv) using large cup forceps (Radial Jaw 4, Boston Scientific, Natick, Massachusetts, USA). All HIV-1 positive patients underwent a total colonoscopy and retrograde ileoscopy for at least 10 cm of distal ileum with conventional or slim scope (model CF or PCF-160 AI, Olympus Medical Europe GmbH, Hamburg, Germany). We obtained specimens (2 biopsies from each site) from the terminal ileum, cecum, ascending, transverse, and descending colon. T cell phenotype and activation markers were analyzed on freshly isolated peripheral blood mononuclear cells (PBMCs) and lamina propria lymphocytes (LPLs) and T cell phenotype and cytokine expression were evaluated after overnight cell culture. One aliquot each of PBMCs and LPLs was stored as dried pellets for RNA extraction.

### 2.3. Specimen Processing

Peripheral blood samples were collected in tubes containing ethylenediaminetetraacetic acid and plasma were previously separated by centrifugation. Blood was processed to obtain PBMCs by Ficoll gradient centrifugation (Lympholyte, Cedarlane Labs, Hornby, Ontario, Canada). Gut biopsies from each intestine site were pooled and processed. Briefly, biopsies collected in RPMI 1640 were washed twice with EDTA wash media, resuspended, and incubated for 1 hour at room temperature in EDTA solution 5 mM. Supernatant containing intraepithelial lymphocytes was removed, and biopsies were digested by 1 hour incubation at 37°C with 1 mg/mL collagenase (Sigma-Aldrich, Milan, Italy) and 1.5 U DNAse I (Sigma-Aldrich, Milan, Italy), allowing the isolation of LPLs that were filtered through a 70 *μ*m cell strainer.

### 2.4. Cell Cultures

PBMCs and LPLs were seeded at concentrations of 2 × 10^6^ cells/mL and 1.5 × 10^6^ cells/mL, respectively, with RPMI media +20% heat inactivated fetal bovine serum (FBS) and cultured overnight at 37°C and 5% CO_2_ in the presence of medium alone or phorbol myristyl acetate (PMA) (3 ng/mL, Sigma Aldrich, Milan Italy) and ionomycin (1 *μ*g/mL, Sigma Aldrich, Milan, Italy). BD GolgiStop (Becton Dickinson, San Jose, CA, USA) was added to all culture conditions. Cells were collected, washed, and stained for T cell phenotype and cytokine expression.

### 2.5. Monoclonal Antibody and T Cell Phenotyping

Phenotype and activation were evaluated by multiparameter flow cytofluorimetric analysis on freshly isolated PBMCs and LPLs by the following anti-human monoclonal antibodies: CD3-PerCP, CD4^+^-APC-Vio770, CD8^+^-FITC, CD45RO-PE-Vio770, CD27-VioBlue, CD38-APC, and HLA-DR-PE (Miltenyi Biotec, Bergisch Gladbach, Germany).

Cultured cells were fixed, permeabilized (BD Cytofix/Cytoperm, Becton Dickinson, San Jose, CA, USA), and stained with combinations of fluorochrome-labeled monoclonal antibodies: CD3-PerCP, CD4-APC-Vio770, CD8-FITC, CD45RO-PE-Vio770, CD27-VioBlue, IFN-*γ*-APC, and IL-17A-PE (Miltenyi Biotec, Bergisch Gladbach, Germany). CD3^+^CD4^+^ cells expressing IFN-*γ* or IL-17A were identified as Th1 and Th17, respectively; CD3^+^CD8^+^ cells expressing IFN-*γ* or IL-17A were identified as Tc1 and Tc17, respectively; CD3^+^CD4^+^ and CD3^+^CD8^+^ expressing both IFN-*γ* and IL-17 were defined as CD4^+^ DP and CD8^+^ DP T cells, respectively.

T cell subpopulations were identified according to the following phenotypic combinations: CD27^+^CD45RO- (naïve), CD27^+^CD45RO^+^ (central memory), and CD27^−^CD45RO^+^CD4^+^ (effector memory) cells [13].

Acquisitions were performed on Miltenyi Biotec flow cytometer-MACSQuant Analyzer (8 fluorescence channels, 3 lasers) and data were analyzed using MACSQuantify software 2.5 (Miltenyi Biotec, Bergisch Gladbach, Germany) with the same gating strategy applied to all samples.

**Figure 1:**
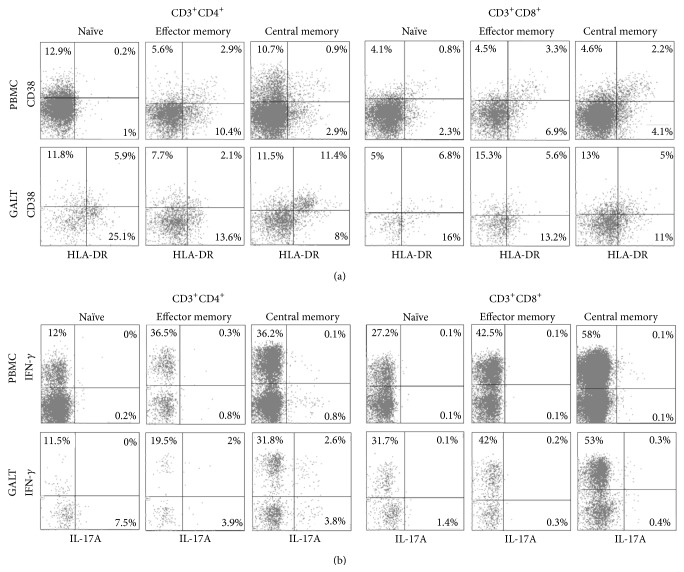
Activation markers, IFN-*γ* and IL-17A expression in CD4^+^ and CD8^+^ T cells subsets in blood and in gut. Panel (a): representative flow cytometry plots outlining CD38 and HLA-DR expression on naïve, effector, and central memory CD4^+^ and CD8^+^ lymphocytes derived from peripheral blood mononuclear cells (PBMCs) and gut-associated lymphoid tissue (GALT). Panel (b): representative flow cytometry plots outlining IFN-*γ* and IL-17A intracitoplasmatic expression on naïve, effector, and central memory CD4^+^ (Th1 and Th17) and CD8^+^ (Tc1 and Tc17) lymphocytes derived from PBMCs and GALT.

At least 100,000 and 10,000* events* in the CD3^+^ lymphocyte gate were analyzed for PBMCs and LPLs, respectively. Representative flow cytometry plots outlining activation markers, IFN-*γ* and IL-17A expression in CD4^+^ and CD8^+^ T cells subsets in blood (PBMCs) and in gut (GALT), are reported in Figure 1.

### 2.6. Virological Analysis

Plasma samples were analyzed for HIV-1 RNA copy number by VERSANT HIV-1 RNA 1.0 kPCR assay (Siemens) with a detection limit of 37 copies/mL.

### 2.7. TaqMan-Based Real-Time RT-PCR Assays for mRNA Expression

Quantitative real-time PCR for IFN-*α*, IFN-*β*, and IFNR1 was carried out with the LightCycler 480 instrument (Roche, Basel, Switzerland). Briefly, total RNA was extracted from PBMCs and LPLs using the RNeasy Plus Universal Tissue Mini Kit (Invitrogen, Carlsbad, CA, USA) and reverse transcribed using the High-Capacity cDNA Reverse Transcription Kit (Applied Biosystem), according to the manufacturer's protocol. Primers and probes for each gene were added to the Probes Master Mix (Roche, Basel, Switzerland) at 500 and 250 nM, respectively, in a final volume of 20 *μ*L. The housekeeping gene *β*-glucuronidase [14] was used as an internal control. Gene expression values were calculated by the comparative Ct method. The primers and probe sequences used for IFN-*α* (Hs. PT.58.24294810.g), IFN-*β* (Hs. PT.58.39481063.g), and IFNR1 (Hs. PT.58.25402720.g) were purchased from Integrated DNA Technologies (IDT), Iowa, USA.

### 2.8. Statistical Analysis

Statistical analyses and graphic presentation were done using SPSS software, version 20.00 (IBM, Somers, NY, USA) on data obtained from PBMC and gut samples of 10 HIV-1 positive patients. In particular, peripheral and intestinal districts were compared by Wilcoxon test for paired samples. Results are given as medians, ranges, and percentages. Linear regression with Spearman's correlation coefficient was used to evaluate correlations between quantitative variables. Differences were considered statistically significant when *p* < 0.05.

## 3. Results

### 3.1. Participant Characteristics

All study participants were Caucasian men with a median age of 42 years (22–53 years). They initiated therapy during chronic infection and had been on ART for a median of 6 years (IQR, 1.75 to 16.25 years); pretherapy median CD4^+^ cell count was 255 cells/mm^3^ (IQR, 42.75 to 406.75 cells/mm^3^), and HIV-1 RNA copies median value was 5.0 Log/mL (IQR, 4.81 to 5.61 Log/mL). All subjects had been virologically suppressed (<37 HIV-1 RNA copies/mL) for at least 1 year at the time of gut biopsy; their median CD4^+^ cell count was 674 cells/mm^3^ (IQR, 564 to 824 cells/mm^3^).

### 3.2. Th17 and Th1 Frequencies in Peripheral Blood and GALT

**Figure 2:**
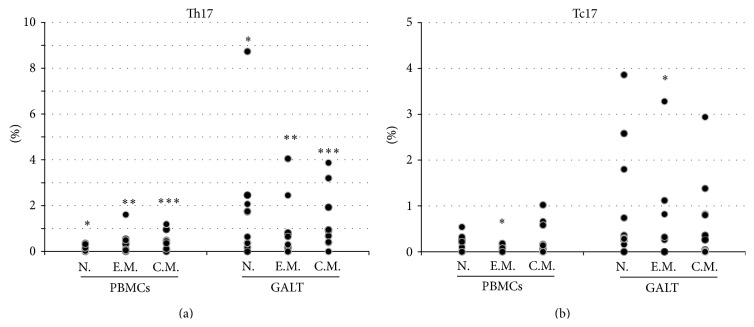
Th17 and Tc17 frequencies in lymphocytes subsets derived from peripheral blood mononuclear cells (PBMC) and gut-associated lymphoid tissue (GALT). Dot plots represent the frequencies of Th17 and Th1 cells in naïve (N), effector memory (E.M.), and central memory (C.M.) CD4^+^ and CD8^+^ in PBMC and in GALT (Wilcoxon test for paired samples *∗*, *∗∗*, and *∗∗∗* = *p* < 0.05).

We compared the Th17 and Th1 frequencies in peripheral blood and GALT samples collected from treated HIV-1-infected patients who had shown a virological response to ART therapy. As seen from Table 1, the median frequencies of Th17, naïve Th17, and CD4^+^ DP T cells were higher in GALT than in peripheral blood (resp., *p* = 0.007, *p* = 0.017, and *p* = 0.008). Furthermore, a trend toward a higher median frequency of Th1 in GALT than in peripheral blood was observed (*p* = 0.059, Table 1). Similar results were obtained comparing the frequencies of central memory and effector memory CD4^+^ T cell subpopulations in the peripheral and GALT compartments. In particular, the median frequencies of both central and effector memory Th17 cell subsets were significantly higher in GALT than in peripheral blood (*p* = 0.013 and *p* = 0.037, resp.). The individual values of Th17 frequencies for naïve and memory subsets are plotted in Figure 2. The same trend was observed for the central and effector memory Th1 cell subsets although the differences did not reach statistical significance (Table 1). Lastly, we found that the median frequencies of both central and effector memory CD4^+^ DP T cells were statistically higher in GALT than in peripheral blood (resp., *p* = 0.011 and *p* = 0.028, Table 1).

### 3.3. Tc17 and Tc1 Frequencies in Peripheral Blood and GALT

Having observed a higher frequency of Th17/Th1 cells in the GALT of HIV-1-infected patients compared to that measured in the peripheral blood, we analyzed the Tc17 and Tc1 frequencies in the same compartments. As seen from Table 1, the median frequencies of Tc17, naïve Tc1, and CD8^+^ DP T cells were significantly higher in GALT than in peripheral blood (resp., *p* = 0.018, *p* = 0.017 and *p* = 0.005, Table 2). A trend towards a higher median frequency of naïve Tc17 cells in GALT than in peripheral blood was also found (*p* = 0.059, Table 2). In contrast, no statistical differences were observed for the frequencies of Tc1, central memory Tc17, central memory Tc1, or central memory CD8^+^ DP T subsets between GALT and peripheral blood. Furthermore, we found that the median frequencies of effector memory Tc17 cells were significantly higher in GALT than in peripheral blood (*p* = 0.035, Table 2) while the median frequencies of effector memory Tc1 and CD8^+^ DP T cells were similar in the two compartments analyzed. The individual values of Tc17 frequencies for naïve and memory subsets are plotted in Figure 2.

### 3.4. CD4^+^ and CD8^+^ T Cell Activation in Peripheral Blood and GALT

**Figure 3:**
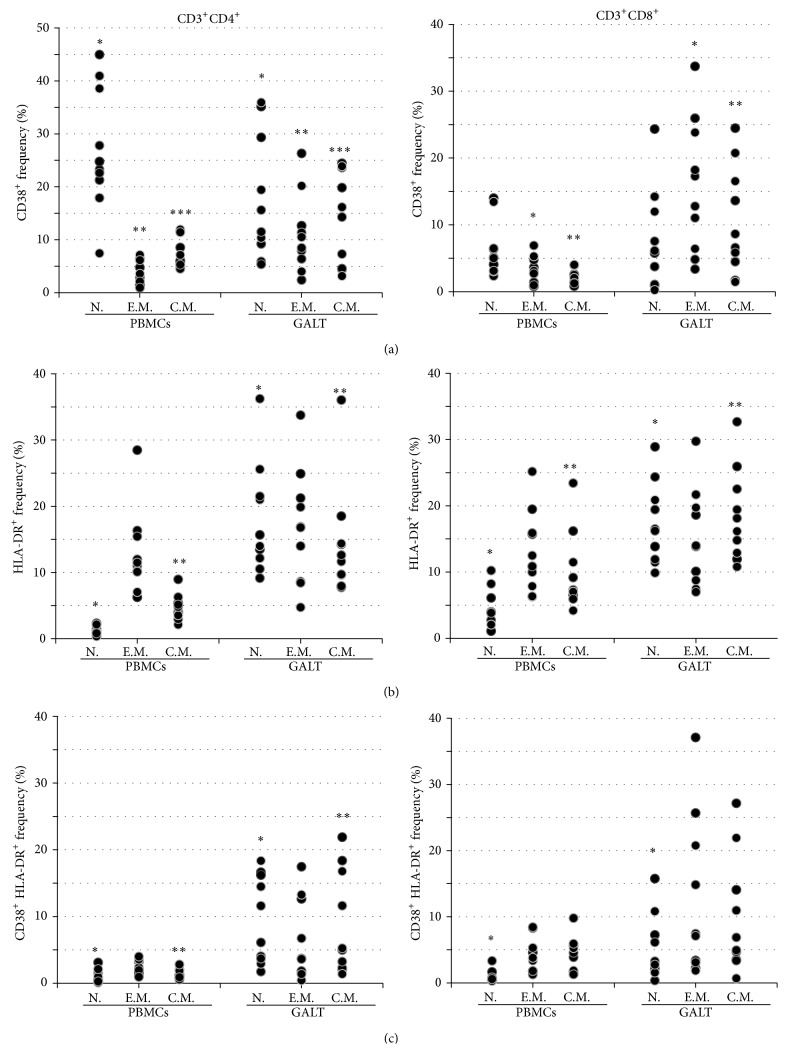
CD38 and HLA-DR expression in lymphocytes subsets derived from peripheral blood mononuclear cells (PBMCs) and gut-associated lymphoid tissue (GALT). Dot plots represent the frequencies of expression of CD38 (Panel (a)) HLA- DR (Panel (b)) and CD38 and HLA-DR (Panel (c)) in naïve (N), effector memory (E.M.), and central memory (C.M.) CD4^+^ and CD8^+^ in PBMC and in GALT (Wilcoxon test for paired samples *∗*, *∗∗*, and *∗∗∗* = *p* < 0.05).

To estimate the activation of T cells in peripheral blood and intestine, we analyzed the frequencies of CD4^+^ and CD8^+^ T cells expressing HLA-DR, CD38 and coexpressing CD38 and HLA-DR. The results are reported in Figure 3. The median frequencies of CD4^+^ cells expressing HLA-DR and coexpressing HLA-DR and CD38 were higher in GALT than in peripheral blood (13.55 versus 4.38: *p* = 0.005, and 4.85 versus 1.35: *p* = 0.005 resp.); these differences seemed to be driven by HLA-DR, as no statistically significant differences were observed with CD38 alone (10.6 versus 14.9, *p* = 0.203). Similar statistical differences in expression of HLA-DR antigen between intestine and peripheral blood were also observed in the central memory and naïve subsets of CD4^+^ T cells (14.85 versus 1.87: *p* = 0.005, and 12.15 versus 4.40: *p* = 0.005 resp.). Furthermore, we found that the frequencies of CD4^+^ T cells expressing CD38 antigen were significantly higher in GALT than in peripheral blood within the effector memory and central memory subpopulations (9.51 versus 3.26: *p* = 0.017, and 15.24 versus 6.61: *p* = 0.047, resp.). By contrast, within the CD4^+^ T cells with naïve phenotype, the expression of CD38 antigen was higher in peripheral blood than in intestine (24.05 versus 13.58: *p* = 0.028). Moreover, median frequencies of naïve and central memory CD4^+^ T cells coexpressing HLA-DR and CD38 were higher in GALT than in peripheral blood (8.87 versus 0.88: *p* = 0.005, and 5.12 versus 1.24: *p* = 0.005, resp.).

As far as the analysis of CD8^+^ T cells expressing HLA-DR and CD38 and coexpressing CD38 and HLA-DR is concerned, the frequencies of CD8^+^ T cells expressing CD38 antigen were higher in gut than in peripheral blood (9.50 versus 2.86: *p* = 0.028); the central memory and effector memory CD8^+^ T subpopulations also expressed more CD38 in intestine than in peripheral blood (7.64 versus 3.73: *p* = 0.013, and 15.04 versus 2.94: *p* = 0.005, resp.). We also observed that the frequencies of CD8^+^ T cells expressing HLA-DR antigen were higher in GALT than in peripheral blood (14.31 versus 8.41: *p* = 0.013); the central memory and naïve CD8^+^ T subpopulations also expressed more CD38 in GALT compared to that in peripheral blood (17.14 versus 7.19: *p* = 0.013, and 16.36 versus 3.87: *p* = 0.005, resp.). The naïve CD8^+^ T subpopulation coexpressing HLA-DR and CD38 was higher in GALT than in peripheral blood (1.6 versus 0.35: *p* = 0.013) (Figure 3).

### 3.5. Relationship between Type I IFN Activation and the Frequencies of Th1, Tc1, Th17, Tc17, CD4^+^, and CD8^+^ T Cells Activated in Peripheral Blood and GALT Compartments

**Figure 4:**
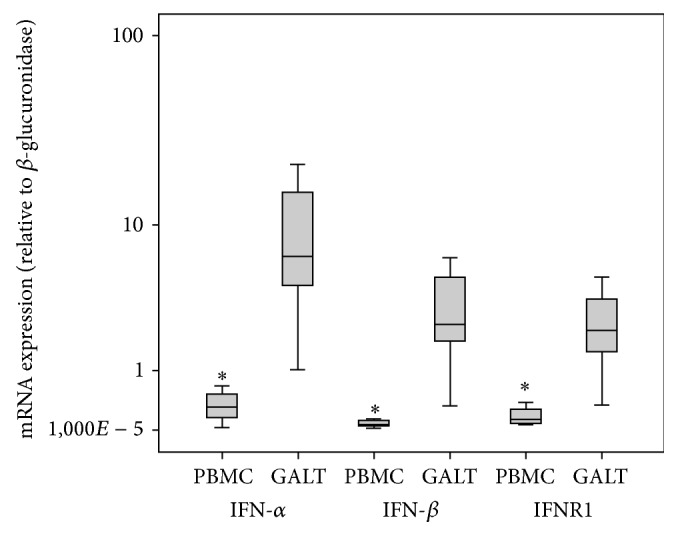
Comparison of the levels of interferon (IFN)-*α*, IFN-*β*, and IFN receptor- (R1-) mRNA in the gut-associated lymphoid tissue (GALT) and peripheral blood mononuclear cells (PBMCs) collected from treated HIV-1-infected patients who had achieved viral load suppression (<37 HIV-1 RNA copies/mL) in response to ART therapy. IFN-*α* (GALT* versus* PBMCs) *p* = 0.01; IFN-*β* (GALT* versus* PBMCs) *p* = 0.01; IFNR1 (GALT* versus* PBMCs) *p* = 0.01. Wilcoxon test was used to compare IFN-*α*, IFN-*β* and IFNR1 in GALT and PBMC.

**Figure 5:**
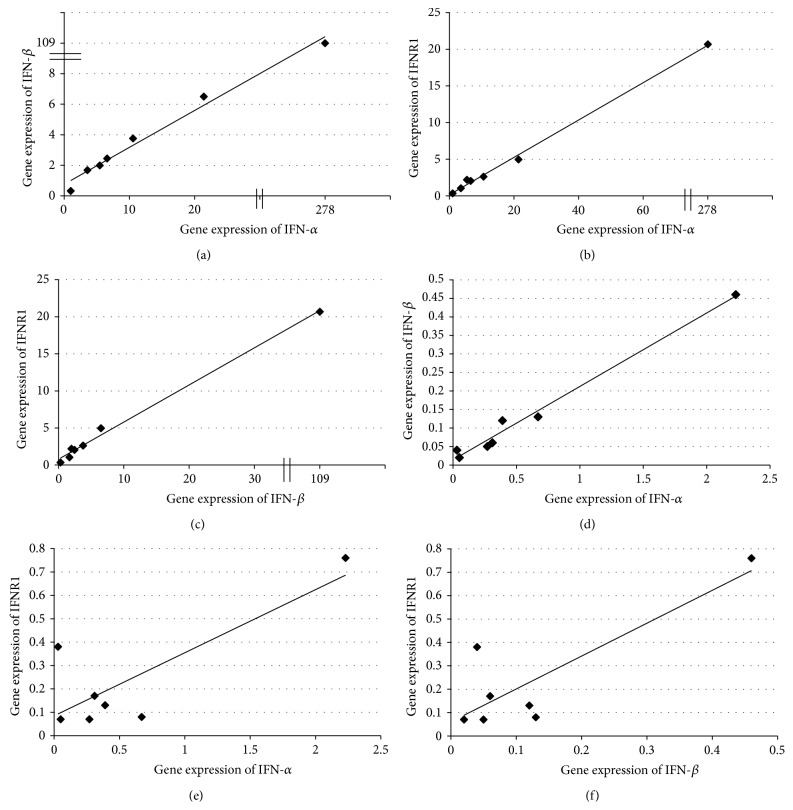
Correlation among type I interferon (IFN) components in the gut-associated lymphoid tissue (GALT) and peripheral blood mononuclear cells (PBMCs) collected from treated HIV-1-infected patients who had achieved viral load suppression (<37 HIV-1 RNA copies/mL) in response to ART therapy. GALT: Panel (a), *r* = 0.98, *p* < 0.001, [IFN-*α* versus IFN-*β*]; Panel (b), *r* = 0.96, *p* < 0.001 [IFN-*α* versus IFN receptor (R1)]; Panel (c), *r* = 0.96, *p* < 0.001 [IFN-*β* versus IFNR1]. PBMCs: Panel (d), *r* = 0.96, *p* < 0.001 [IFN-*α* versus IFN-*β*]; Panel (e), *r* = 0.32, *p* = 0.48 [IFN-*α* versus IFN receptor (R1)]; Panel (f), *r* = 0.50, *p* = 0.25, [IFN-*β* versus IFNR1]. Spearman's rho coefficient was used to assess the correlation between type I IFN components in GALT and PBMCs.

Next, we evaluated whether there was any relationship between endogenous type I IFN production and the frequencies of naïve and memory Th1/Th17 and Tc1/Tc17 cells and those of CD4^+^ and CD8^+^ T cells expressing HLA-DR, CD38 and coexpressing CD38 and HLA-DR. First, we examined the IFN-*α*, IFN-*β*, and IFNR1 gene expression in PBMCs and GALT collected from HIV-1-infected patients who achieved a virological suppression in response to ART. Results indicated that transcript levels of IFN-*α*, IFN-*β*, and IFNR1 are extremely variable in both PBMCs and GALT [coefficient of variation (CV) > 100%]. However, we found that median values of IFN-*α*, IFN-*β*, and IFNR1-mRNAs were, respectively, 21-, 40-, and 16-fold higher in GALT compared to those measured in PBMCs (Figure 4, IFN-*α*:  *p* = 0.01; IFN-*β*:  *p* = 0.01; IFNR1: *p* = 0.01). A coordinated activation among type I IFN members was observed in GALT as evidenced by the strong correlations between IFN-*α* and IFN-*β* and between IFNR1 and IFN-*α*/*β* subtypes (Figures 5(a)–5(c)). By contrast, IFN-*α* and IFN-*β* were correlated to each other but not to IFNR1 in PBMCs (Figures 5(d)–5(f)). Having observed a highly coordinated expression of type I IFN components in GALT compared to that measured in PBMCs, we evaluated whether there was any correlation between type I IFN activation and the frequencies of naïve or memory Th1/Tc1 and Th17/Tc17 cell subsets. We observed that IFN-*α*/*β* mRNA levels were negatively related to the frequencies of both IFN-*γ*-producing CD4^+^ T and CD8^+^ T naïve cells and also of IFN-*γ*-producing CD8^+^ memory T cell subsets (central memory and effector memory) in GALT (Table 3). In addition, negative correlations between the IFNR1 levels and the frequencies of both IFN-*γ*-producing CD4^+^ T naïve cells and the frequencies of IFN-*γ* CD4^+^ central memory and CD8^+^ effector memory T cells were also recorded in GALT. By contrast, there was no relationship between type I IFN response and IFN-*γ*-producing CD8^+^ or CD4^+^ T cell subsets (naïve, central memory, and effector memory) in the peripheral blood compartment (data not shown). As far as the analysis of the relationship between IFN-*α*, IFN-*β*, and IFNR1 production and naïve and memory Th17/Tc17 response is concerned, there was no significant relationship between type I IFN activation and Th17/Tc17 response measured in either GALT or peripheral blood compartments (data not shown). Lastly, we evaluated whether there was any correlation between type I IFN expression and the frequencies of CD4^+^ and CD8^+^ T cells expressing HLA-DR, CD38 and coexpressing CD38 and HLA-DR. We found that the amount of IFN-*α* (*r* = 0.82, *p* = 0.023) and IFN-*β* (*r* = 0.81, *p* = 0.022), but not that of IFNR1, measured in GALT, was correlated with frequencies of CD4^+^ T cells expressing CD38 in peripheral blood. Furthermore, levels of IFN-*α* and IFN-*β* in peripheral blood were correlated to both the frequencies of CD4^+^ T cells coexpressing CD38 and HLA-DR (IFN-*α*: *r* = 0.96, *p* = 0.0001; IFN-*β*: *r* = 0.92, *p* = 0.003) and those of CD8^+^ effector memory T cells expressing HLA-DR measured in GALT (IFN-*α*: *r* = 0.85, *p* = 0.014; IFN-*β*: *r* = 0.78, *p* = 0.36). Conversely, no relationship was recorded between levels of type I IFN components and the frequencies of CD4^+^ and CD8^+^ T cells expressing HLA-DR, CD38 and coexpressing CD38 and HLA-DR measured in the same compartment (data not shown).

## 4. Discussion

The gut mucosa is known to be a compartment where the interplay between HIV-1 and the host's immune system takes center stage. Therefore, we examined the frequency, phenotype, and functional status of T cell subpopulations in the GALT and peripheral blood of virologically suppressed HIV-1-infected patients on ART therapy, focusing on the IFN-*γ* or IL-17^+^ subset of CD4^+^ T and CD8^+^ T cells. Throughout the long antiretroviral treatment, we observed a persistent immune activation in GALT expressed by high levels of CD4^+^/HLA-DR^+^ and CD4^+^CD38^+^/HLA-DR^+^ with higher levels of Th17 and Tc17 with respect to peripheral blood. In this regard, it has been reported that gut Th17 cells had a much greater capacity to produce proinflammatory cytokines than did those from the blood, but this capacity was dramatically reduced from the earliest stages of HIV infection [15]. Furthermore, Nigam et al. reported that Tc17 and Th17 cells were present in all lymphoid and gastrointestinal tissues studied with predominance in small intestine [16].

Our results also confirm that prolonged antiretroviral treatment improves GALT immune function despite the persistence of immune activation. In this context, we speculate on the importance of discovering new therapeutic strategies that are able to delete immune activation. In fact, the detection of Th17 after PMA stimulation emphasizes that unexhausted cells are present in GALT despite the continuous status of immune activation. Our previous study demonstrated that eight months of ART increased intestinal CD4^+^ and Th17 cells and reduced levels of T cell activation and proliferation and the magnitude of intestinal CD4^+^ T cell reconstitution correlated with the reduction of plasma LPS [17]. It would be useful to know if Th17 recovered in GALT occurs through routes other than ART. In our opinion, the type I IFN response plays a central role in the pathogenesis of HIV-1 infection both by restricting viral replication and spread and by contributing to chronic immune activation which is currently considered the driving force of acquired immunodeficiency syndrome [18]. This is supported by some degree of correlation found between levels of type I IFN components and the activation of CD4^+^ and CD8^+^ T cells. Thus, to gain new insights into the Th1/Tc1 and Th17/Tc17 response in long- term treated HIV-1-infected patients, we also evaluated the expression of IFN-*α* and IFN-*β* and IFNR1 and their relationships with T cell subsets in GALT and peripheral blood compartments. Interestingly, a more vigorous and coordinated IFN-*α*/*β* and IFNR1 response was observed in mucosal gut compared to that measured in the peripheral blood of HIV-1-infected patients. These findings could indicate an incomplete suppression type I IFN immune activation in GALT despite the long-term therapy, which could be linked to the persisting mucosal viral burden. In agreement with the above observation, Macal et al. demonstrated that long-term therapy could reduce, but not fully suppress, HIV-RNA levels in the gut mucosa, although substantial gut CD4^+^ T cell restoration was still possible [19]. Limited information is available on the expression of type I IFN pathways in the gastrointestinal tract of HIV-1-infected patients. Previous studies examining gene expression responses in GALT to acute-stage and chronic-stage HIV-1 infection in ART-naïve patients found a broad increase in IFN-driven immune responses [20–22]. The clinical significance of an upregulation of type I IFN components in GALT compared to the peripheral compartment in long-term treated HIV-1-infected patients remains unknown. However, a significant increase in mucosal gene expression regulating IFN immune activation and inflammation was detected in chronically HIV-1-infected patients with high viral loads but not in long-term nonprogressor patients, supporting the evidence of a detrimental role for IFN-*α*/*β* during HIV-1 infection [21]. Furthermore, previous studies found a correlation between incomplete mucosal CD4^+^ T cell restoration and increased immune activation in HIV infection despite ART [19, 23, 24]. Here, we observed that IFN-*α*/*β*/mRNA levels were negatively related to the frequencies of IFN-*γ*-producing CD8^+^ T cell subsets (naïve, central memory, and effector memory) in GALT suggesting that the activation of type I IFN response could have a negative impact on CD8^+^ T cell responses. A negative relationship between type I IFN response and IFN-*γ*-producing CD4^+^ naïve T cells was also recorded. It is currently unknown whether these negative correlations between type I IFN components and CD8^+^ T cell subsets and Th1 naïve cells can be considered positive or negative in terms of HIV-1-associated enteropathogenesis. However, it has been reported that viral suppression in long-term nonprogressor patients may result, in part, from efficient maintenance of CD4^+^ T helper and HIV-1-specific CD8^+^ T cell responses in both GALT and circulating lymphocytes [21].

As far as the analysis of type I IFN activation and Th17/Th1 response was concerned, we did not found any relationships between type I IFN activation and Th17/Tc17 response in either compartment analyzed. By contrast, a negative impact of type I IFN on Th17 differentiation in multiple sclerosis [25] or on the secretion of Th17-polarizing cytokines in human dendritic cells [26] has recently been proposed. However, as a variety of signals can block Th17 commitment including that of IFN-*γ*, the highly upregulated type I IFN components in GALT could negatively influence the production of IFN-*γ*, thereby contributing indirectly to the restoration of Th17 during ART therapy. Further studies are urgently needed to characterize the controversial role of type I IFN in HIV-1 disease and its relationship with IFN-*γ* or IL-17A-producing CD4^+^ T or CD8^+^ T cell subsets in the gastrointestinal tract of HIV-1-infected patients.

All these findings would be greatly strengthened by the evaluation of soluble markers of inflammation (LPS, scCD14, IL-6, and D-dimers) and their correlation with Th17, Th1, Tc17, Tc1 frequencies, and type I IFN components. Another important analysis is the expression of ISGs and other IFN-related genes to better characterize IFN activation pathways in the peripheral blood and GALT compartments of HIV-1 patients. Unfortunately, the material collected in the present study was only enough for the current experiments.

In conclusion, our comparative investigation of the frequency, phenotype, and functional status of Th1/Tc1 and Th17/Tc17 cell populations in intestinal mucosa and peripheral compartments of long-term HIV-1-infected patients provides new insights into the role of mucosal pathology in HIV-1 disease progression. A significant increase in the gene expression of type I IFN components was detected in GALT compared to peripheral blood. This was negatively related to IFN-*γ*-producing CD8^+^ T cell subsets and also to IFN-*γ*-producing CD4^+^ naïve T cells but not to the Th17/Tc17 response. A better understanding of the mucosal relationship between the Th1/Th17 and Tc1/Tc17 axis and antiviral innate defenses during HIV-1 infection will shed more light on the mechanisms of HIV-1 enteropathogenesis.

## Figures and Tables

**Table 1:** Th17 and Th1 frequencies in peripheral blood and gut-associated lymphoid tissue (GALT) samples collected from HIV-1-infected patients (*n* = 10) who achieved virological suppression in response to ART therapy.

Frequencies	GALT	Peripheral blood	*p* values
Th17 cells (median/range)	1.06/0.21–3.66	0.26/0.00–0.67	*p* = 0.007
Th1 cells (median/range)	5.71/0.23–25.19	0.16/0.01–28.72	*p* = 0.059
CD4^+^ DP T cells (median/range)	0.17/0.0–2.28	0.005/0.0–0.12	**p** = 0.017
Naïve Th17 cells (median/range)	1.19/0.0–8.73	0.09/0.0–0.36	**p** = 0.008
Naïve Th1 cells (median/range)	1.01/0.23–11.56	0.04/0.0–13.0	*p* = 0.79
Naïve CD4^+^ DP T cells (median/range)	0.0/0.0–0.49	0.0/0.0–0.05	*p* = 0.69
Central memory Th17 cells (median/range)	0.935/0.0–3.87	0.24/0.00–1.19	**p** = 0.013
Central memory Th1 cells (median/range)	7.65/0.19–33.14	0.22/0.0–36.21	*p* = 0.169
Central memory CD4^+^ DP T cells (median/range)	0.15/0.0–3.15	0.01/0.0–0.14	**p** = 0.011
Effector memory Th17 cells (median/range)	0.48/0.0–4.05	0.21/0.00–1.61	**p** = 0.037
Effector memory Th1 cells (median/range)	3.28/0.00–19.53	0.33/0.0–47.04	*p* = 0.959
Effector memory CD4^+^ DP T cells (median/range)	0.16/0.0–2.03	0.0/0.0–0.34	**p** = 0.028

^*∗*^Wilcoxon test for paired samples was used to compare Th17 and Th1 frequencies in peripheral blood and GALT. Significant values are highlighted in bold.

**Table 2:** Frequencies of Tc17 and Tc1 in peripheral blood and gut-associated lymphoid tissue (GALT) samples collected from HIV-1-infected patients (*n* = 10) who achieved virological suppression in response to ART therapy.

Frequencies	GALT	Peripheral blood	*p* values
Tc17 cells (median/range)	0.21/0.00–1.50	0.03/0.00–0.1	**p** = 0.018
Tc1 cells (median/range)	10.59/0.32–56.34	0.36/0.0–58.24	*p* = 0.114
CD8^+^ DP T cells (median/range)	0.095/0.0–1.16	0.005/0.0–0.11	**p** = 0.017
Naïve Tc17 cells (median/range)	0.17/0.0–1.9	0.11/0.0–0.27	*p* = 0.059
Naïve Tc1 cells (median/range)	6.24/0.37–41.74	0.49/0.0–31.61	**p** = 0.005
Naïve CD8^+^ DP T cells (median/range)	0.0/0.0–0.41	0.01/0.0–0.07	*p* = 0.262
Central memory Tc17 cells (median/range)	0.16/0.0–1.47	0.05/0.00–0.51	*p* = 0.169
Central memory Tc1 cells (median/range)	15.38/0.52–58.75	1.36/0.0–72.9	*p* = 0.386
Central memory CD8^+^ DP T cells (median/range)	0.09/0.0–1.17	0.04/0.0–1.17	*p* = 0.063
Effector memory Tc17 cells (median/range)	0.14/0.0–1.64	0.025/0.00–0.09	**p** = 0.035
Effector memory Tc1 cells (median/range)	10.27/0.27	0.45/0.0–83.54	*p* = 0.720
Effector memory CD8^+^ DP T cells (median/range)	0.00/0.0–1.24	0.00/0.00–1.64	*p* = 0.721

^*∗*^Wilcoxon test was used to compare Tc17 and Tc1 frequencies in peripheral blood and GALT. Significant values are highlighted in bold.

**Table 3:** Analysis of the relationship between type I interferon (IFN) activation and naïve and memory Th1 or Tc1 cell subsets measured in the gut-associated lymphoid tissue (GALT) of HIV-1-infected patients.

	IFN-*γ* CD4^+^ naïve T cell	IFN-*γ* CD4^+^ central memory T cell	IFN-*γ* CD4^+^ effector memory T cell	IFN-*γ* CD8^+^ naïve T cell	IFN-*γ* CD8^+^ central memory T cell	IFN-*γ* CD8^+^ effector memory T cell
IFN-*α*	**r** = −0.92	*r* = −0.71	*r* = −0.50	**r** = −0.80	**r** = −0.80	**r** = −0.89
	**p** = 0.003	*p* = 0.071	*p* = 0.25	**p** = 0.036	**p** = 0.036	**p** = 0.007

IFN-*β*	**r** = −0.92	*r* = −0.71	*r* = −0.50	**r** = −0.80	**r** = −0.80	**r** = −0.89
	**p** = 0.003	*p* = 0.071	*p* = 0.25	**p** = 0.036	**p** = 0.036	**p** = 0.007

IFNR1	**r** = −0.85	**r** = −0.78	*r* = −0.57	*r* = −0.75	*r* = −0.75	**r** = −0.85
	**p** = 0.014	**p** = 0.036	*p* = 0.18	*p* = 0.052	*p* = 0.052	**p** = 0.014

^*∗*^Spearman's rho coefficient was used to assess the correlation between type I IFN activation and naïve and memory Th1 or Tc1 cell subsets. Significant correlations are highlighted in bold.
